# Removal of slow-pulsing artifacts in in-phase ^15^N relaxation dispersion experiments using broadband ^1^H decoupling

**DOI:** 10.1007/s10858-018-0193-2

**Published:** 2018-06-02

**Authors:** Soumya Deep Chatterjee, Marcellus Ubbink, Hugo van Ingen

**Affiliations:** 1 Macromolecular Biochemistry, Leiden Institute of Chemistry, Leiden University, P.O Box 9502, 2300 RA Leiden, The Netherlands; 2 Present Address: NMR Group, Bijvoet Center for Biomolecular Research, Utrecht University, Padualaan 8, 3584 CH Utrecht, The Netherlands

**Keywords:** Relaxation dispersion, Decoupling, Protein dynamics, Composite pulse

## Abstract

Understanding of the molecular mechanisms of protein function requires detailed insight into the conformational landscape accessible to the protein. Conformational changes can be crucial for biological processes, such as ligand binding, protein folding, and catalysis. NMR spectroscopy is exquisitely sensitive to such dynamic changes in protein conformations. In particular, Carr–Purcell–Meiboom–Gill (CPMG) relaxation dispersion experiments are a powerful tool to investigate protein dynamics on a millisecond time scale. CPMG experiments that probe the chemical shift modulation of ^15^N in-phase magnetization are particularly attractive, due to their high sensitivity. These experiments require high power ^1^H decoupling during the CPMG period to keep the ^15^N magnetization in-phase. Recently, an improved version of the in-phase ^15^N-CPMG experiment was introduced, offering greater ease of use by employing a single ^1^H decoupling power for all CPMG pulsing rates. In these experiments however, incomplete decoupling of off-resonance amide ^1^H spins introduces an artefactual dispersion of relaxation rates, the so-called slow-pulsing artifact. Here, we analyze the slow-pulsing artifact in detail and demonstrate that it can be suppressed through the use of composite pulse decoupling (CPD). We report the performances of various CPD schemes and show that CPD decoupling based on the 90_*x*_–240_*y*_–90_*x*_ element results in high-quality dispersion curves free of artifacts, even for amides with high ^1^H offset.

**Electronic supplementary material:**

The online version of this article (10.1007/s10858-018-0193-2) contains supplementary material, which is available to authorized users.

## Introduction

Biological macromolecules such as nucleic acids and proteins are non-rigid entities that can populate a variety of conformers in their energy landscape (Frauenfelder et al. 1991; Wolynes 2005; Henzler-Wildman and Kern 2007). The lowest energy conformation, the ground state, is often able to transiently access higher-energy conformations. Even when their population is low (< 10%) and life times is short (~ ms), these excited states can be crucial for biologically important processes such as enzyme catalysis (Hammes 1964; Eisenmesser et al. 2005; Henzler-Wildman et al. 2007; Palmer 2015; Kim et al. 2017), ligand binding or protein–protein interactions (Sugase et al. 2007; Schneider et al. 2015; Pratihar et al. 2016; Xiao et al. 2016; Zhao et al. 2017; Delaforge et al. 2018), and protein folding (Korzhnev et al. 2010; Neudecker et al. 2012; Kimsey et al. 2015; Libich et al. 2015; Franco et al. 2017; Culik et al. 2018). While these states cannot be detected directly due to their transient and lowly populated nature, NMR experiments (Akke and Palmer 1996; Fawzi et al. 2010; Kovermann et al. 2016) are uniquely able to provide a detailed, atomistic description of the energy landscape. In particular, relaxation dispersion and chemical exchange saturation transfer experiments are particularly powerful herein, as they give access to the population, life times and structures of excited states (Palmer et al. 2001; Vallurupalli et al. 2012, 2017; Sauerwein and Hansen 2015; Xue et al. 2015; Lisi 2016; Massi and Peng 2018; Gopalan et al. 2018).

In Carr–Purcell–Meiboom–Gill (CPMG) relaxation dispersion experiments, the characterization of the minor state is derived from the major state peaks by measurement of their effective transverse relaxation rate *R*_2,*eff*_, as a function of the pulsing rate in the CPMG period. Signals of nuclear spins that experience exchange between states with different chemical shifts are affected by exchange-induced line broadening, an effect that depends on the free precession interval (2τ_cp_) between the refocusing pulses in the CPMG element (Palmer et al. 2001; Sauerwein and Hansen 2015). Analysis of the resulting relaxation dispersion curve, a plot of the *R*_2,*eff*_ versus CPMG frequency (1/4τ_cp_), allows determination of the rate of exchange (*k*_ex_), population of minor state (*p*_b_) and the absolute chemical shift difference (|*Δϖ*|) between the exchanging states. Importantly, since the shape of the dispersion profile depends on *Δϖ*, data is typically acquired at two fields to accurately determine the exchange parameters (Sauerwein and Hansen 2015).

The ^15^N backbone amide spin is the most popular nucleus for CPMG RD experiments, due to the simplicity of isotope-labeling, the straightforwardness of the two-spin ^1^H–^15^N spin system, and the high sensitivity and resolution afforded by these experiments. A critical aspect of these experiments is appropriate handling of differences in the intrinsic *R*_2_ of the in-phase (*N*_*x,y*_) and anti-phase (2*N*_*x,y*_*H*_*z*_) ^15^N magnetization which are generated in the free evolution periods. Anti-phase terms have higher intrinsic relaxation rates due to a contribution of ^1^H spin flips to their decay. The original implementation of the ^15^N CPMG RD experiment uses a relaxation-compensation scheme to average the *N*_*x,y*_ and 2*N*_*x,y*_*H*_*z*_ relaxation rates (Loria et al. 1999b). The ^15^N CPMG sequence of Hansen et al. (2008b) (CW–CPMG) measures the dispersion profile of pure in-phase *N*_*x,y*_ by applying high-power continuous wave (CW) ^1^H decoupling during the CPMG train, offering enhanced sensitivity for non-deuterated proteins. Recently, Jiang et al. (2015) modified this sequence (ST–CW–CPMG) to use a single CPMG train with the Yip and Zuiderweg phase cycle (2004) and a single CW decoupling power, yielding dispersion curves free of off-resonance artifacts for a wider range of ^15^N offset frequencies.

Both CW–CPMG sequences are nevertheless sensitive to artifacts from ^1^H off-resonance effects (Hansen et al. 2008b; Yip and Zuiderweg 2004). Amide ^1^H spins that are far off-resonance from the CW decoupling field are not fully decoupled from the ^15^N spin, resulting in generation of 2*N*_*x,y*_*H*_*z*_ magnetization through the residual *J*-coupling. Consequently, higher *R*_2,*eff*_ values will be measured for low *ν*_CPMG_ values, for which free precession periods are long and more of the antiphase terms will be generated. This so-called slow-pulsing artifact shows up as an artefactual dispersion curve, interfering with accurate extraction of minor-state parameters.

Here, we analyze the slow-pulsing artifact in ^15^N CW–CPMG sequences in detail and demonstrate a simple method for its removal. In that, we took inspiration from the work of Chakrabarti et al. (2016), where composite pulse decoupling (CPD) was used to suppress ^1^H off-resonance effects in exchange mediated saturation transfer experiments. We investigated the performance of various CPD schemes in CW–CPMG sequences and demonstrate here that high power CPD based on the 90_*x*_–240_*y*_–90_*x*_ element achieves artifact-free dispersion curves over a wide range of ^1^H offsets.

## Materials and methods

### NMR samples

NMR experiments were recorded on a sample of 2.5 mM uniformly ^15^N/^13^C-labelled Cu(II) azurin in 25 mM potassium phosphate buffer at pH 5.49 with 5% D_2_O. Labelled azurin was produced and purified according to a previously published protocol with modifications for incorporating ^13^C-glucose and ^15^N-ammoniumchloride (Karlsson et al. 1989).

### NMR experiments

Relaxation dispersion experiments, using the ST–CW–CPMG sequence, were recorded at 298 K on Bruker Avance III HD spectrometers operating at 850 and 950 MHz ^1^H Larmor frequency and equipped with TCI cryoprobes. The constant-time CPMG relaxation delay (*T*_relax_) was set to 40 ms with *ν*_CPMG_ set to 25, 50, 75, 100, 125, 175, 225 (2×), 275, 300, 350, 400 (2×), 500, 550, 600, 650, 700 (2×), 750, 800 (2×), 850, 900, 950 and 1000 Hz respectively, run in an interleaved manner. Duplicates were used to estimate the error in *R*_2,*eff*_. The errors were set to 0.2 s^−1^ at minimum. The pulse length of the ^15^N refocusing pulses in the CPMG train was 90 µs. For ^1^H decoupling, either CW decoupling or a CPD-scheme (GARP, DIPSI, MLEV16, WALTZ16, 90_*x*_–240_*y*_–90_*x*_) was used. This was implemented by changing the “cw:f1” statement in the pulse program to read “cpds1:f1” (pulse program available upon request). In either case, the decoupling field strength was 14.7 kHz (17 µs ^1^H 90° pulse), applied at 8.2 ppm ^1^H offset. A total of 3072/120 points were acquired in the ^1^H/^15^N dimension with an acquisition time of 90/27.85 ms and a relaxation delay of 2 s and 4 scans per FID. A reference spectrum, without the relaxation delay, was also recorded. NMR data were processed with NMRPipe (Delaglio et al. 1995), using linear prediction in the ^15^N dimension and Lorentz-to-Gauss window functions. Peak volumes were obtained by peak fitting using FuDa (Hansen, http://www.biochem.ucl.ac.uk/hansen/fuda/), and subsequently converted into effective relaxation rates via *R*_2,*eff*_(*ν*_CPMG_) = − 1/*T*_relax_·ln(*I* (*ν*_CPMG_)/*I*_0_), where *I*_0_ is the peak intensity in a reference spectrum recorded without the relaxation delay *T*_relax_. The *R*_2,*eff*_ values measured using the ST–CW–CPMG sequence were corrected for *R*_1_-contribution according to the formula described by Jiang et al. (2015) using an estimate of 0.95 s^−1^ *R*_1_- and 10.5 s^−1^ for *R*_2_-contribution for all residues. Dispersion curves obtained with either CW or CPD decoupling were compared by calculating the RSMD between the curves for all residues:

$$RMSD=~\sqrt {\frac{1}{N}\mathop \sum \limits_{{i=1}}^{N} {{\left( {R_{{2,eff}}^{{CPD,i}} - R_{{2,eff}}^{{CW,i}}} \right)}^2}}$$

where *i* is the index of a particular ν_CPMG_ value and the summation runs over the *N* recorded points, equal to the number of points per dispersion curve (*M*) times the number of residues. The systematic difference between the CW or CPD-based dispersion curves was calculated from the average point-by-point difference per residue and is tabulated in Table S1. To compensate for these systematic differences, an “*R*_2_-offset compensated” RMSD was calculated by replacing the CPD-based *R*_2,*eff*_ values with the offset compensated values:

$$R_{{2,eff}}^{{CPD,compensated,i}}=R_{{2.eff}}^{{CPD,i}} - \frac{1}{M}\mathop \sum \limits_{{i=1}}^{M} \left( {R_{{2,eff}}^{{CPD,i}} - R_{{2,eff}}^{{CW,i}}} \right)$$

### Simulation of ^15^N CW–CPMG dispersion profiles

To evaluate the magnitude of the slow-pulsing artifact in relaxation dispersion profiles, numerical simulations of a two-spin ^1^H–^15^N system were carried out, assuming a non-deuterated protein. The evolution of magnetization in this spin system was calculated for the CPMG part of the CPMG–CW and CPMG–ST–CW sequence, including the flanking ^15^N 90° pulses. Simulations in the absence of exchange and neglecting pulse imperfections were performed in operator space by solving the complete homogeneous master equation as described by Allard et al. (1998) and Helgstrand et al. (2000) using the open source computing language GNU Octave (http://www.gnu.org/software/octave/) (Eaton et al. 2008). All simulation used the parameters detailed below unless noted otherwise. The ^15^N spin was assumed to be on-resonance. The magnetic field strength was set to 19.9 T, corresponding to ^1^H Larmor frequency of 850 MHz. Relaxation rates were calculated using overall rotational correlation time τ_c_ of 9 ns, a value of 0.85 for the squared generalized order parameter, 100 ps for the correlation time for internal motions, and − 172/+10 ppm for the ^15^N/^1^H chemical shift anisotropy. Relaxation due to neighboring protons was included as described in ref. (Allard et al. 1998) by including a virtual proton at 1.85 Å, resulting in *R*_2_ values of in-phase and anti-phase ^15^N magnetizations of 13.6 and 26.7 s^−1^ respectively. Dispersion experiments were simulated with *T*_relax_ set to 40 ms, and *ν*_CPMG_ values ranging from 25 to 1000 Hz, the ^15^N 180° refocusing pulse was set to 90 µs, ^1^H CW decoupling field strength was set to 14.7 kHz (17 µs ^1^H 90°).

## Results

Measurement of in-phase ^15^N CPMG relaxation dispersion profiles critically relies on ^1^H decoupling to measure the pure in-phase *N*_*x,y*_ relaxation rate without contamination by the anti-phase relaxation rate. As pointed out in the work of Jiang et al. (2015), the decoupling field strength has a practical limit of roughly 14 kHz, resulting in a residual *J* coupling interaction for amide protons at non-zero offset to the decoupling field. This interaction causes slow interconversion of in-phase and anti-phase magnetization during the CPMG period, which will lead to undesired averaging of the in-phase and anti-phase relaxation rates (Loria et al. 1999b; Hansen et al. 2008b). To first approximation, this averaging can be described by the equation derived by Palmer et al. (1992) for calculating the effective relaxation rate in spin echo sequences. Here, it is adapted and reformulated to express to the size of the slow-pulsing artifact *A*:

1 $$A=\frac{1}{2}\left( {R_{2}^{{anti}} - R_{2}^{{in}}} \right)\left( {1 - {\text{sinc}}\pi {J_r}2{\tau _{cp}}} \right)$$

where *R*_2_^*in*^ and *R*_2_^*anti*^ are the ^15^N in-phase and anti-phase transverse relaxation rates, *J*_*r*_ is the residual *J*-coupling, and 2*τ*_cp_ is the inter-pulse delay in the CPMG pulse train. In the limit of perfect decoupling J_r_ ≈ 0, the sinc factor approaches 1 and *A* ≈ 0 for all *τ*_cp_ values. For non-zero *J*_*r*_, *A* approaches zero in the limit of fast pulsing where *τ*_cp_ is very small. For slow pulsing, however, there is a non-zero artifact, with a theoretical limit of 0.5 (*R*_2,*anti*_ − *R*_2,*in*_) for infinitely slow pulsing. In practice, *J*_r_ can be as much 16 Hz (for 3 ppm ^1^H offset at 850 MHz) and 2*τ*_cp_ is typically at most 20 ms, which would generate a maximum artifact of roughly 10% of the difference between the anti-phase and in-phase relaxation rate.

**Fig. 1:**
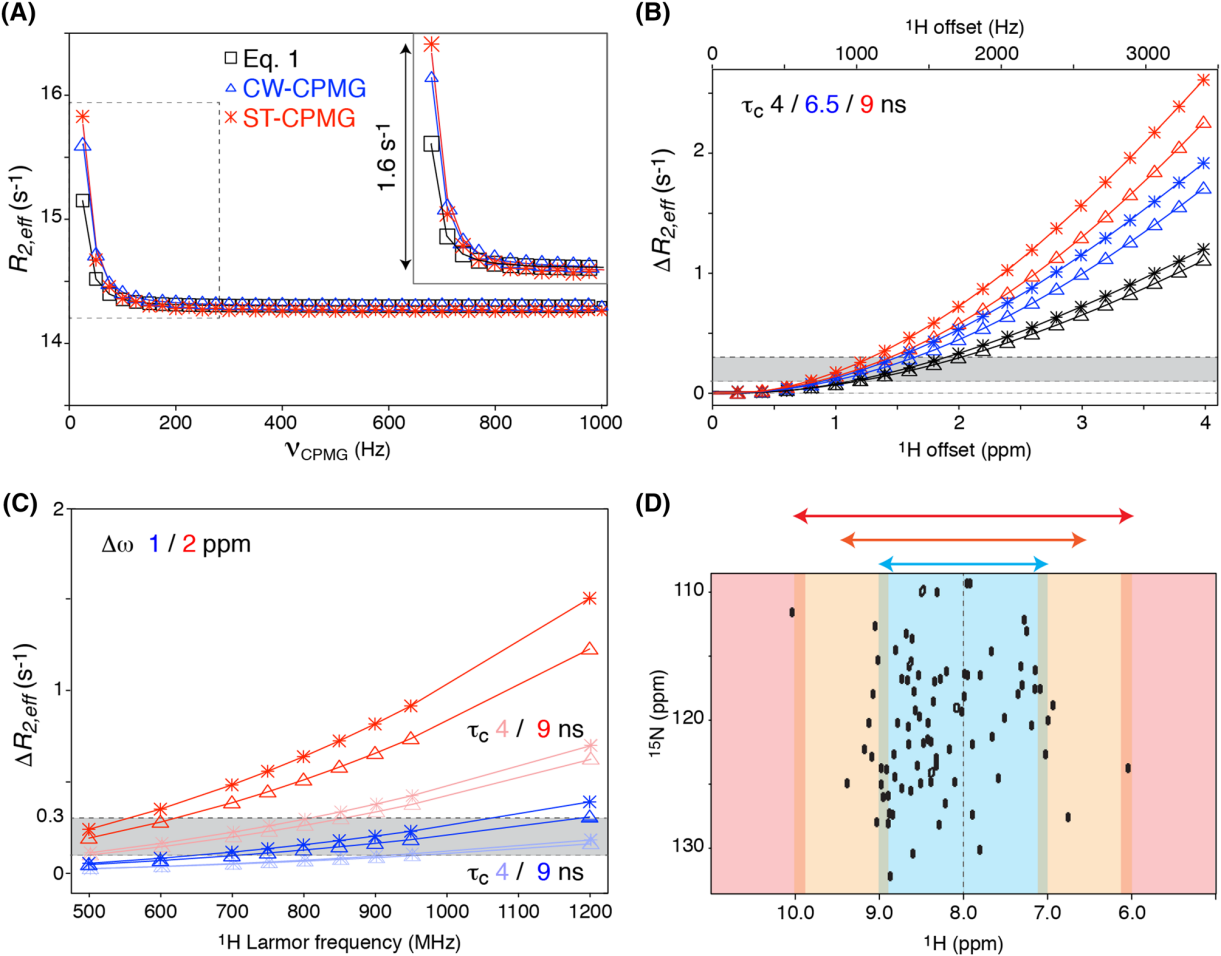
Impact of the slow-pulsing artifact on simulated relaxation dispersion profiles. **a** Simulated slow-pulsing artifact caused by incomplete *J*_NH_ decoupling in the CW–CPMG and the ST–CW–CPMG implementation of the in-phase ^15^N CPMG experiment. Solid lines are fits obtained using the program CATIA (Hansen, http://www.biochem.ucl.ac.uk/hansen/catia/) assuming two-site exchange. The artifact expected based on Eq. 1 is shown for comparison. The boxed region is expanded in the inset. The ^1^H offset from the decoupling field was set to 3 ppm, assuming an 850 MHz spectrometer. **b, c** Maximum size of the artifact (Δ*R*_2,*eff*_) as a function of (**b**) ^1^H offset for proteins of 4, 6.5 and 9 ns correlation times at 850 MHz; **c** magnetic field strength for 1 and 2 ppm ^1^H offset for proteins of 4 and 9 ns tumbling times. The gray area indicates the typical experimental error in range of 0.1–0.3 s^−1^. **d** The typical accessible ^1^H offset ranges, color coded into a ^15^N–^1^H HSQC spectrum. Assuming the ^1^H CW field is centered at 8 ppm, the blue region is accessible up to the highest magnetic fields, orange is accessible up to 600 MHz, and the red region is inaccessible. In **a**–**c** simulated profiles are shown for both CW–CPMG (open triangle) and ST–CPMG (asterisk) pulse sequences; color coding indicated in the figure. All simulations are based on a non-exchanging N–H spin system. Simulation parameters are given in “Materials and Methods” section, unless noted otherwise

To assess more precisely how the slow pulsing artifact is manifested in ^15^N CPMG–CW and ST–CW experiments, numerical simulations of these sequences were performed in Liouville space for a non-exchanging two spin N–H system. Figure 1a compares the obtained dispersion profiles for the two experiments with the predicted curve based on Eq. 1, for a N–H system with 3 ppm ^1^H offset at an 850 MHz spectrometer. Whereas a flat curve is expected for a non-exchanging system, systematically increased *R*_2,*eff*_ values are measured in the slow pulsing regime for both pulse sequences. While Eq. 1 is derived for periods of free evolution in absence of a decoupling field, the curvature of the slow-pulsing artifact matches the predicted sinc dependence on the pulsing rate. The size of the artifact is somewhat underestimated by Eq. 1. The original CW sequence shows slightly lower sensitivity to the artifact than the ST–CW experiment. This difference can be traced back to presence of the ^15^N refocussing pulse in between the two halves of the total CPMG period in the CW experiment. Importantly, since the shape of the artifact is virtually indistinguishable from a *bona-fide* dispersion profile, the artefactual *R*_2,*eff*_ values can be fitted to an actual dispersion curve (see solid lines in Fig. 1a), illustrating the potential impact on the extracted exchange parameters.

Since the size of the slow pulsing artifact is governed by the relaxation difference between in-phase and anti-phase magnetization, it is dependent on protein size. Large proteins have more efficient ^1^H–^1^H spin flips which increase the anti-phase relaxation rate. Figure 1b compares the magnitude of the artifact for three different protein sizes as function of ^1^H offset from the decoupling field. For larger proteins, where the chance of finding amide protons at high offset is also higher, the artifact can be well above 1 s^−1^. At offsets larger than ~ 1000 Hz the slow pulsing artifact will be higher than the typical experimental error (on the order of 0.1–0.3 s^−1^) (Hansen et al. 2008a), as also noted by Jiang et al. (2015).

Since relaxation dispersion data need to be acquired at two magnetic fields in order to extract accurate protein dynamics parameters, we compared the size of the slow pulsing artifact for amide groups at 1 and 2 ppm ^1^H offset as function of magnetic field strength in Fig. 1c. High field strengths are not only attractive because of the sensitivity and resolution they afford, but also because they are more sensitive to exchange processes as they increase the frequency difference between states, *Δϖ*. However, for a given resonance, the offset from the decoupling field, and thus the slow pulsing artifact, will increase with increasing magnetic field strength. Strikingly, the artifact will already be significant at 1 ppm offsets for medium-sized proteins in a future 1.2 GHz spectrometer. To illustrate the impact of the slow pulsing artifact, generated by the inability of CW irradiation to decouple the full width of the amide spectrum, the HSQC can be divided in three areas: a narrow region ± ~1 ppm around the carrier frequency of the decoupling field that will be free of significant artifacts, the region beyond ± ~2 ppm in which significant artifacts will already occur at the lowest typical field strength, and the intermediate region (Fig. 1d).

**Fig. 2:**
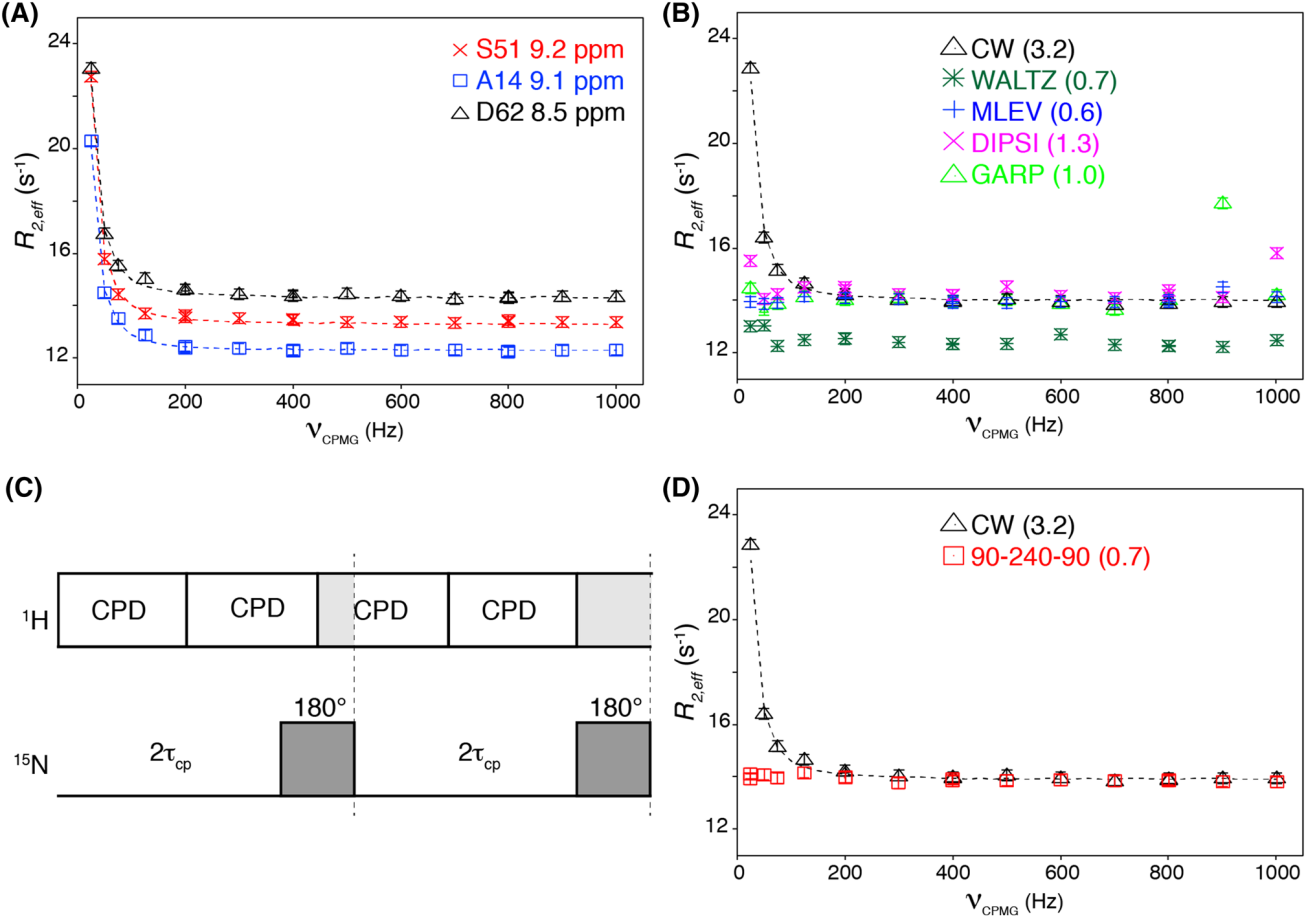
Suppression of slow pulsing artifacts by composite pulse based broadband ^1^H decoupling. **a** Experimental relaxation dispersion curves for three residues measured using the ST–CW–CPMG sequence with the ^1^H decoupling field centered at 16 ppm. Dotted lines are fits to Eq. 1 to guide the eye. **b** Experimental dispersion curves for residue D62 using the indicated ^1^H decoupling schemes, all centered at 16 ppm. **c** When the ^1^H decoupling power is fixed, there is an inevitable timing mismatch between complete ^1^H inversion at the end of each composite pulse block (CPD) and the point (dotted line) of complete ^15^N inversion. As a result, decoupling is incomplete and resulting in elevated *R*_2,*eff*_ values. **d** Experimental dispersion curves for residue D62 using CW and 90_*x*_–240_*y*_–90_*x*_^1^H decoupling schemes, both centered at 16 ppm. In **b, d** between brackets are the average root-mean-square deviation (RMSD) to a straight line over all 114 residues in azurin. Note that the RMSD obtained with CW decoupling centered at 8.2 ppm was 0.7 s^−1^

To confirm the results obtained from simulations, we experimentally demonstrated the problem using the ST–CW–CPMG pulse sequence on a sample containing azurin, a 16 kDa electron transfer metalloprotein (Adman 1991). A small subset of residues in azurin have been reported to undergo conformational exchange on the millisecond timescale (Korzhnev et al. 2003). To emphasize the slow pulsing artifact, we purposely centered the ^1^H decoupling field at 16 ppm such that the dispersion profiles are dominated by the artifact (Fig. 2a). Using this setup, we next screened several broadband decoupling sequences for their ability to suppress the artifact. These sequences rely on composite pulses to offer good population inversion even in the presence of off-resonance effects (Shaka and Keeler 1987), and thus should be able to suppress the artifact in theory. As can be seen in Fig. 2b, a wide range of CPD schemes indeed suppressed the artifact. Notably, the use of GARP (Shaka et al. 1985) and DIPSI2 (Shaka et al. 1988) results in spurious elevated *R*_2,*eff*_ values at high pulsing rates, rendering the dispersion curves unusable. These spikes originate from the timing mismatch between the continuous train of (composite) 180° pulses on the ^1^H channel on the one hand and the repetition of free-evolution and 180° refocusing pulses on the ^15^N channel on the other hand. This mismatch results in incomplete decoupling at the end of each τ_cp_ period and thus elevated *R*_2,*eff*_ values (Fig. 2c). As noted by Jiang et al. (2015), the duration of the mismatch is short when using adequately high power CW ^1^H decoupling, and thus the effect is small. Both DIPSI2 and GARP use particularly long composite pulses (corresponding to the length of 2590° and 1054° rotation, respectively), which aggravates the impact of the timing mismatch, in particular at high pulsing rates, where the effects from each τ_cp_ period are compounded. Indeed, use of WALTZ (540° duration) (Shaka et al. 1983) and MLEV (360° duration) (Levitt and Freeman 1981; Levitt et al. 1982a, b) with shorter duration of the composite pulse did not cause such high spikes. We next applied the 90_*x*_–240_*y*_–90_*x*_ CPD scheme, which was recently used to suppress artifacts from incomplete ^1^H decoupling in exchange mediated saturation transfer experiments (Chakrabarti et al. 2016). The 90_*x*_–240_*y*_–90_*x*_ CPD sequence has a short overall duration (420° rotation) and offers relatively broadband inversion, free from off-resonance effects without relying on supercycles (Levitt et al. 1982a; Levitt 1982). Gratifyingly, the 90_*x*_–240_*y*_–90_*x*_ sequence effectively eliminated the artifacts without causing appreciable spikes or scatter in *R*_2,*eff*_ values (Fig. 2d). The requirement for a short duration of the CPD element also means that the broadband performance of CPD decoupling cannot be used to reduce the decoupling power. Tests showed that reducing the decupling power to 7 kHz (34 µs decoupling pulse) resulted in spurious artifacts dominating the dispersion curves at high CPMG pulsing rates (data not shown).

While successful in suppressing the slow-pulsing artifacts, the use of composite pulse sequences for decoupling results in systematic differences in *R*_2,*eff*_ values compared to those obtained using CW decoupling. This is most apparent from the WALTZ data in Fig. 2b, showing systematically reduced *R*_2,*eff*_ values compared to the CW reference data. Such offsets between the CPD-derived and CW-derived dispersion curve are also found for MLEV and 90_*x*_–240_*y*_–90_*x*_ decoupling, although typically much smaller. When using the 90_*x*_–240_*y*_–90_*x*_ sequence, the average offset over all residues was found to be ~ 0.3 s^−1^ with 90% of the profiles having offsets below 0.6 s^−1^ (see Supplemental Table S1). Since this offset is small and the absolute value of *R*_2,*eff*_ is not of importance when fitting dispersion curves, it will have negligible impact on the usefulness of the data obtained with CPD decoupling schemes.

**Fig. 3:**
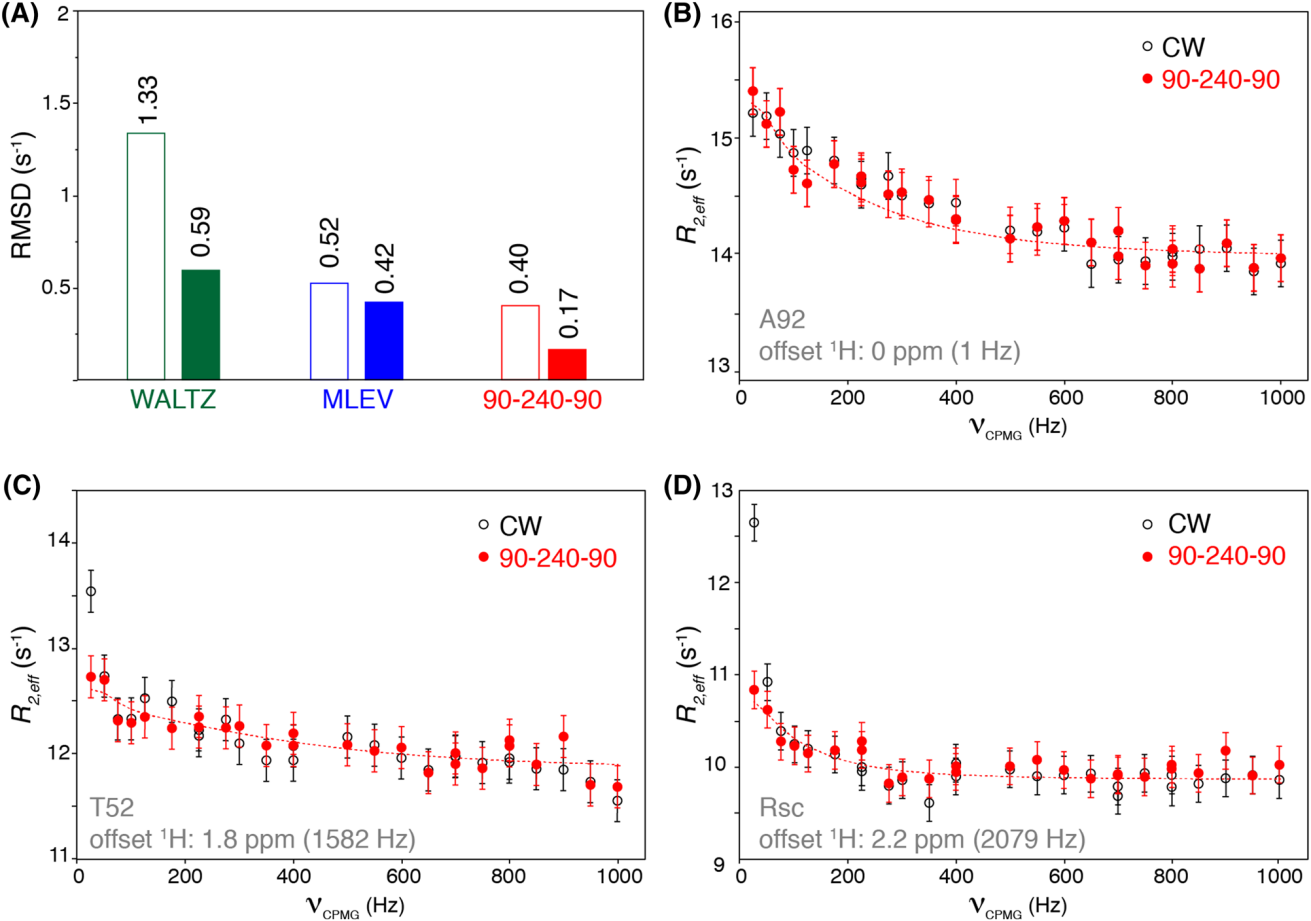
The 90_*x*_–240_*y*_–90_*x*_ decoupling scheme offers high-quality dispersion curves free of slow-pulsing artifact. **a** Average RMSD between CPD- and CW-based dispersion curves over all analyzed residues in azurin. Open/closed bars refer to the RMSD without/with compensating for the offset between the curves. **b**–**d** Experimental dispersion curves for both CW and 90_*x*_–240_*y*_–90_*x*_ based experiments for amide resonances of A92 (no ^1^H offset, panel **b**), and T52, and an unassigned Arg sidechain resonance (Rsc) both with significant ^1^H offset. Data for panels **b, c** recorded at 850 MHz. Data for panel **d** recorded at 950 MHz. Dotted lines are best-fit dispersion curves obtained using CATIA (Hansen, http://www.biochem.ucl.ac.uk/hansen/catia/). The CPD data were corrected for the systematic offset to the CW data before plotting

Having established that WALTZ, MLEV and 90_*x*_–240_*y*_–90_*x*_ decoupling sequences are able to suppress the slow pulsing artifact, we further tested their efficacy in a regular experimental setup with the decoupling field centered at 8.2 ppm. The obtained *R*_2,*eff*_ values were compared point-by-point between the CPD and the CW data-set, and the root-mean-square deviation (RMSD) between data sets was calculated with and without compensating for the systematic offset in *R*_2,*eff*_ values between the two datasets (Fig. 3a). Clearly, the 90_*x*_–240_*y*_–90_*x*_ sequence performs best with an average RMSD to the reference CW data set of 0.17 s^−1^, which is on the order of the experimental error. The high quality of the data is visible from comparison of profiles obtained for residues with negligible ^1^H offset, such as shown in Fig. 3b. At high ^1^H offset from the decoupling field, the CW data suffers from the slow pulsing artifact, which is absent when using the 90_*x*_–240_*y*_–90_*x*_ CPD sequence, as exemplified for T52 in Fig. 3c. Notably, this residue shows the slow pulsing artifact superimposed on a genuine dispersion of *R*_2,*eff*_ values. From the comparison to the CPD-based experiment, it becomes clear that the data point at 25 Hz ν_CPMG_ pulsing rate is strongly affected by the slow pulsing artifact with *R*_2,*eff*_ value spuriously elevated by ~ 1 s^−1^. As a final experiment, we recorded both CW and CPD-based dispersion profiles at the national ultra-high field NMR Facility at 950 MHz. At this field, the resonance with the highest ^1^H offset shows a slow-pulsing artifact of ~ 1.5 s^−1^ in the CW experiment, which is effectively suppressed when using the 90_*x*_–240_*y*_–90_*x*_ decoupling sequence (Fig. 3d).

## Discussion

We have investigated the impact of the slow-pulsing artifact in in-phase ^15^N relaxation dispersion experiments by theoretical considerations, numerical simulations and experiments. We show that the artifact can be removed by using CPD-based ^1^H decoupling during the CPMG period. Out of the tested CPD sequences, the 90_*x*_–240_*y*_–90_*x*_ sequence offers the best performance: the artifact is fully suppressed, while retaining shape of the dispersion curve obtained using CW decoupling within experimental error. Notably, this is done without introducing spurious spikes in *R*_2,*eff*_ values at high pulsing rates, and with minimal offset to the CW-based dispersion profiles. Critical to its performance seems to be short duration of the composite pulse combined with relatively high quality of off-resonance performance.

The cause of the slight offset between the CPD and CW-based dispersion profiles is unclear. Closer inspection shows that the magnitude of the offset shows no correlation to either the N, H_N_, or H_α_ chemical shift and that both reference (no CPMG delay) and dispersion experiment (with CPMG delay) have slightly altered intensities (~ 2–5%) in the CPD experiment compared to the CW experiment. The effect on the reference experiment, where the decoupling block is carried out before the recycle delay, signifies that the both types of decoupling result in a different steady-state magnetization, presumably both for water and protein protons.

As for the water magnetization, a disadvantage of using CPD over CW decoupling is the loss of control over its state. Whereas in the CW case the water magnetization is spin-locked and returned to + *z* after the CPMG period, continuous alteration between *x* and *y*-pulse phase during the 90_*x*_–240_*y*_–90_*x*_ CPD element causes dephasing and loss of water polarization. Experimental tests (Hiller et al. 2005) demonstrate this effect and show that after a 2 s delay, corresponding to the recycle delay to the next proton excitation pulse, there is minimal difference between the water polarization in the CPD and CW case (see Supplemental Fig. S1). Here, radiation damping caused by the high Q of the cryogenic probe likely aids the recovery of the water magnetization in the CPD case. Additionally, the low pH of the sample (5.5) will slow down amide-water exchange and thus additionally dampen the effect of (residual) water saturation.

In the original implementation of the in-phase dispersion experiment described by Hansen et al. (2008b), the strength of the decoupling field is matched to the CPMG pulsing rate to avoid the timing mismatch as indicated in Fig. 2c. In principle, such matching could also be done when using CPD decoupling schemes, which should result in decreased scatter in the dispersion curves. While simulations indeed show such improvement in performance, an experimental test showed a severe increase in scatter, presumably due to a point-to-point variation in the steady state of the water and aliphatic proton magnetization.

As noted in Fig. 1, the slow-pulsing artifact will be particularly problematic at high magnetic field strengths. At such high fields, it may be better to use TROSY–CPMG sequences (Loria et al. 1999a), which do not suffer from the slow-pulsing artifact, even for non-deuterated moderately sized proteins. The relative sensitivity of TROSY and in-phase CPMG experiments is best assessed experimentally as it not only depends on magnetic field strength but also on protein size, labeling pattern, and temperature. Next to the absolute sensitivity, one may also consider that lower ^15^N relaxation rates during the CPMG period allow the use of longer CPMG delays, increasing the sensitivity to slow motions (Loria et al. 1999a), as well as spectral quality of TROSY spectra (reduced overlap vs. presence of anti-TROSY lines). Additionally, in case data at lower field strength have been recorded using the in-phase CPMG experiment it may be necessary to record these at high fields too.

In conclusion, we show here that the use of broadband ^1^H decoupling, in particular using the 90_*x*_–240_*y*_–90_*x*_ sequence, is a viable and attractive option for recording in-phase ^15^N relaxation dispersion data. This option is particularly relevant when the protein spectrum contains resonances far from center. It offers artifact-free dispersion profiles without the need for recording data in multiple sets or the need for eliminating of data points, all without compromising data quality.

## Electronic supplementary material

Below is the link to the electronic supplementary material.

Supplementary material 1 (PDF 145 KB)
