# Supplementary material for: Removal of slow-pulsing artifacts in in-phase 15N relaxation dispersion experiments using broadband 1H decoupling
Source: J Biomol NMR. 2018 Jun 2;71(2):69–77. doi: 10.1007/s10858-018-0193-2 (PMC6061081; doi:10.1007/s10858-018-0193-2)
Supplement: Supplementary file 1 — Supplementary material 1 (PDF 145 KB) [file 10858_2018_193_MOESM1_ESM.pdf]

Supplemental Material

for

Removal of slow-pulsing artifacts in in-phase ^{15}N relaxation dispersion experiments using broadband ^{1}H decoupling

Soumya Deep Chatterjee[1], Marcellus Ubbink[1], Hugo van Ingen[1,2*]

[1]Macromolecular Biochemistry, Leiden Institute of Chemistry,

Leiden University, P.O Box 9502, 2300 RA, Leiden, The Netherlands

[2]current address: NMR Group, Bijvoet Center for Biomolecular Research,

Utrecht University, Padualaan 12, 3854 CH, Utrecht, The Netherlands

* to whom correspondence should be addressed: h.vaningen@uu.nl, +31-30-253 99 39

Table S1. $R_{2,eff}$ offset between CPD and CW-derived dispersion curves[a].

CPD scheme	*average* (s^{-1})	*range* (s^{-1})	*90% limit* (s^{-1})[b]
90_x-240_y-90_x	-0.27	-1.13 – 0.74	0.57
MLEV	-1.24	-6.77 – 1.06	2.92
WALTZ	-0.85	-4.44 – 1.15	1.80

[a] data recorded with offset of decoupling field centered at 8.2 ppm. Negative offset values indicate that CPD-derived $R_{2,eff}$ values are lower than in the CW-based experiment.

[b] absolute values.

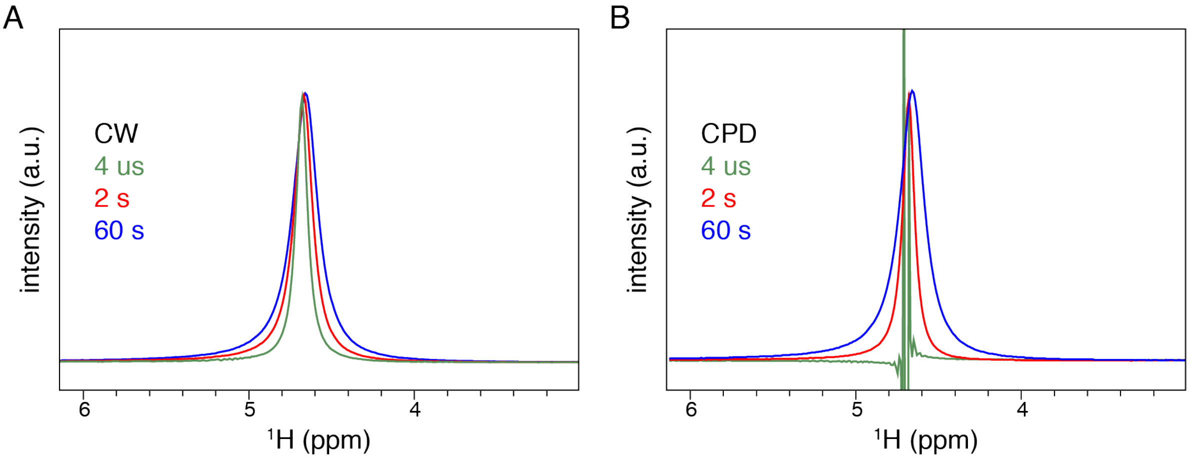

Figure S1: Measurement of water polarization when using either CW (A) or 90_x-240_y-90_x CPD decoupling (B). The following pulse sequence was used: water-selective excitation – CW/CPD decoupling for 40 ms – water flip back – variable delay (4 us: green; 2 s: red; 60 s: blue) – gradient – tap pulse (0.1 us). Apart from the gradient and tap pulse used here to readout the water polarization,[1] this scheme is used as a heat compensation block in the reference experiment right before the recycle delay (2 sec in our experiments) and first excitation pulse. While the CW scheme retains the majority of the water polarization, the 90_x-240_y-90_x scheme results in water dephasing and loss of water polarization, resulting in distorted signal with low integral value. After 2 sec recovery, the majority of the polarization is recovered.

Supplemental references

1. Hiller, S., Wider, G., Etezady-Esfarjani, T., Horst, R. & Wüthrich, K. Managing the solvent water polarization to obtain improved NMR spectra of large molecular structures. *Journal of Biomolecular NMR* ***32,*** *61–70 (2005).*
